# Interaction of removal Ethidium Bromide with Carbon Nanotube: Equilibrium and Isotherm studies

**DOI:** 10.1186/2052-336X-12-17

**Published:** 2014-01-08

**Authors:** Omid Moradi, Mehdi Norouzi, Ali Fakhri, Kazem Naddafi

**Affiliations:** 1 Department of Chemistry, Shahre-Qods Branch, Islamic Azad University, Tehran, Iran; 2 Department of Virology, School of Genetics, School of Public Health, Tehran University of Medical Sciences, Tehran, Iran; 3 Department of Environmental Health Engineering, School of Public Health and Center for Air Quality Research, Institute for Environmental Research, Tehran University of Medical Sciences, Tehran, Iran

**Keywords:** Adsorption, Ethidium bromide, Single-walled carbon nanotube, Isotherm

## Abstract

Drinking water resources may be contaminated with Ethidium Bromide (EtBr) which is commonly used in molecular biology laboratories for DNA identification in electrophoresis. Carbon nanotubes are expected to play an important role in sensing, pollution treatment and separation techniques. In this study adsorption of Ethidium Bromide on single-walled carbon nanotubes (SWCNTs) and carboxylate group functionalized single-walled carbon nanotube (SWCNT-COOH) surfaces have been investigated by UV–vis spectrophotometer. The effect of contact time, initial concentration and temperature were investigated. The adsorbents exhibits high efficiency for EtBr adsorption and equilibrium can be achieved in 6 and 3 min for SWCNTs and SWCNT-COOH, respectively. The effect of temperature on adsorption of EtBr by toward adsorbents shows the process in this research has been endothermic. The results showed that the equilibrium data were well described by the Langmuir isotherm model, with a maximum adsorption capacity of 0.770 and 0.830 mg/g for SWCNTs and SWCNT-COOH, respectively. The adsorption of EtBr on SWCNT-COOH is more than SWCNTs surfaces. A comparison of kinetic models was evaluated for the pseudo first-order, pseudo second-order models. Pseudo second-order was found to agree well with the experimental data.

## Introduction

The contamination of drinking water with Non-radioactive materials such as Ethiduim bromide (EtBr) has become one of the most serious problems in water environment, especially in urban areas [1-3]. Ethidium bromide (3,8-diamino-6-phenyl-5-ethylphenanthridinium bromide, EtBr;), a powerful mutagenic [4], is an intercalating agent which resembles a DNA base pair. Due to its unique structure, it can easily intercalate into DNA strand. Therefore, it is commonly used as nucleic acid fluorescent tag in various techniques of the life science field. EtBr is a potent mutagen for which the Environmental Health & Safety advises a detoxification protocol [5], since earlier recommended oxidation by household bleach was recognized to lead to possible byproducts that could be more hazardous than the EtBr itself [6]. Taking the adsorption of dyes and organic pollutant, the usability of various natural and synthetic adsorbents have been studied, such as banana peel [7], orange peel [7], calcined layered double hydroxides [8], hypercrosslinked polymeric adsorbent [9], pinecone derived activated carbon [10]. Usually, the effectiveness of any adsorption process largely depends on the physicochemical properties of the adsorbent used. Since the discovery by Iijima [11], carbon nanotubes (CNTs) have attracted great attention in multidisciplinary areas due to their unique hollow tube structure and their many outstanding mechanical, electronic and optical properties [12]. In particular, carbon nanotubes (both multi-walled (MWCNT) and single walled (SWCNT)) are promising materials for several applications such as high performance composites [13-15], components in water filters [16,17], environmental sensors [18,19], building blocks for electronic nanodevices [20], drug delivers [21], among others. CNTs are also considered to be extremely good adsorbents and successfully remove many kinds of organic and inorganic pollutants such as pentachlorophenol [22], benzene, toluene, ethylbenzene and p-xylene [23], o-xylene and p-xylene [24] and heavy metal ions such as U(VI) [25], Cr(VI) [26], Zn(II) [27], Cu(II) [28], Pb(II) [29], Hg(II) [30] and Cd(II) [31,32] from water. Activation of CNTs plays an important role in enhancing maximum adsorption capacity. Activation causes modification in the morphology and functional groups surfaces and causes removal of amorphous carbon. The objective of this study was to investigate the possible use of single walled carbon nanotubes as an alternative adsorbent material for the removal of EtBr from drinking water. Adsorption isotherms and kinetics parameters were also calculated and discussed. The dynamic behavior of adsorption was investigated on the effect of initial EtBr concentration, contact times and temperature of the solution.

## Material and methods

SWCNTs and SWCNT-COOH (Armchair (6,6), Young’s Modulus (0.94 T TPa), Tensile strength (GPa126.2 T), purity *>* 95%; diameter 1–2 nm; length, 5–30 nm; surface area, ~ 400 m^2^*/*g; and manufacturing method purchased from NanoAmor Nanostructured & Amorphous Materials, Inc. (USA) and catalytic chemical vapor deposition (CVD)) were prepared from DC-PECVD (Model: SI-PE80) Toseye Hesgarsazan Asia Co. Doubly distilled water was used and all adsorbents were washed before using. A scanning electron microscope (SEM) (JEOL JSM-5600 Digital Scanning Electron Microscope) was used to characterize the SWCNT-COOH and SWCNTs for morphological information. Ethidium bromide (molecular Weight: 394.35; molecular Formula: C_21_H_20_BrN_3_) was supplied by Merck, Germany (maximum purity available, ≥95%). All solutions were prepared with deviations of less than ±0.1% from the desired concentrations.

### Adsorption experiments

The determination of ethiduim bromide concentration was performed using Lambda-EZ150UV/Vis Spectrophotometer at a wavelength of 274 nm. The blank used in the experiment was distilled water without any EtBr. Batch adsorption experiments were performed in glass bottles with EtBr solution (1 L) of the prescribed concentration ranging from 10 to 40 mg/L and 20 mg of SWCNTs or SWCNT-COOH was added to each bottle. The amount of SWCNTs or SWCNT-COOH was fixed in all experimental steps. All experiments were conducted by mixing 20 mL of aqueous solutions with 0.02 g of the adsorbent solution composed of SWCNTs with EtBr, solution No. 1 was called and solution composed of SWCNTCOOH with EtBr, No.2 was called. Then, the suspension 1 and 2 were centrifuged at 5000 rpm for 5 min and 2 min, respectively. The amount of the EtBr adsorbed onto the adsorbent was determined by the difference between the initial and remaining concentration of EtBr solution. The adsorption capacity of EtBr on adsorbent was calculated using the following equation [33]:

(1) $$q_{e}=\frac{\left(C_{i}-C_{t}\right)V}{W}$$

where C_i_ is the initial EtBr concentration and C_t_ is the EtBr concentration (mg*/*L) at any time, V is the volume of solution (L) and W is the mass of the adsorbents (g). The data analysis was carried out using correlation analysis employing least square method and the average relative error (ARE) is calculated using the following equation [33]:

(2) $$\mathrm{ARE}\left(\%\right)=\frac{100}{n}\;\sum_{i}^{n}\left|\frac{q_{i},\mathrm{cal}-q_{i},\exp}{q_{i},\exp}\right|$$

where N is the number of data points. Each experiment was conducted in triplicate under identical conditions to confirm the results, and was found reproducible (experimental error within 3%).

### Equilibrium

To optimize the design of an adsorption system for the adsorption, it is important to establish the most appropriate correlation for the equilibrium curves. Various isotherm equations have been used to describe the equilibrium nature of adsorption. Some of these isotherms are Langmuir and Freundlich. One of the most common isotherm models which are widely used is the Langmuir model. It is observed that the Langmuir isotherms can be linearized to at least four different types. The Langmuir isotherm model can be expressed as:

(3) $$q_{e}=\frac{1}{\left(1+\mathrm{KCe}\right)}$$

where Q_m_ (mg/g) and K (L/mg) are Langmuir constants related to adsorption capacity and energy of adsorption, respectively [34-36]. The essential characteristics of the Langmuir isotherm can be expressed in terms of a dimensionless equilibrium parameter (R_L_) which can be defined by:

(4) $$R_{L}=\frac{1}{1+\mathrm{KCO}}$$

**Figure 1:**
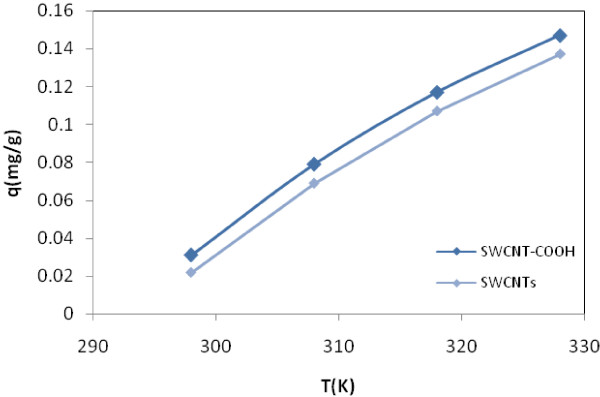
**Effect of temperature on the adsorption of EtBr with SWCNTs and SWCNT-COOH, initial concentration, 20 ml, 30 mg** ***/*** **L; adsorbent dosage, 20 mg.**

**Figure 2:**
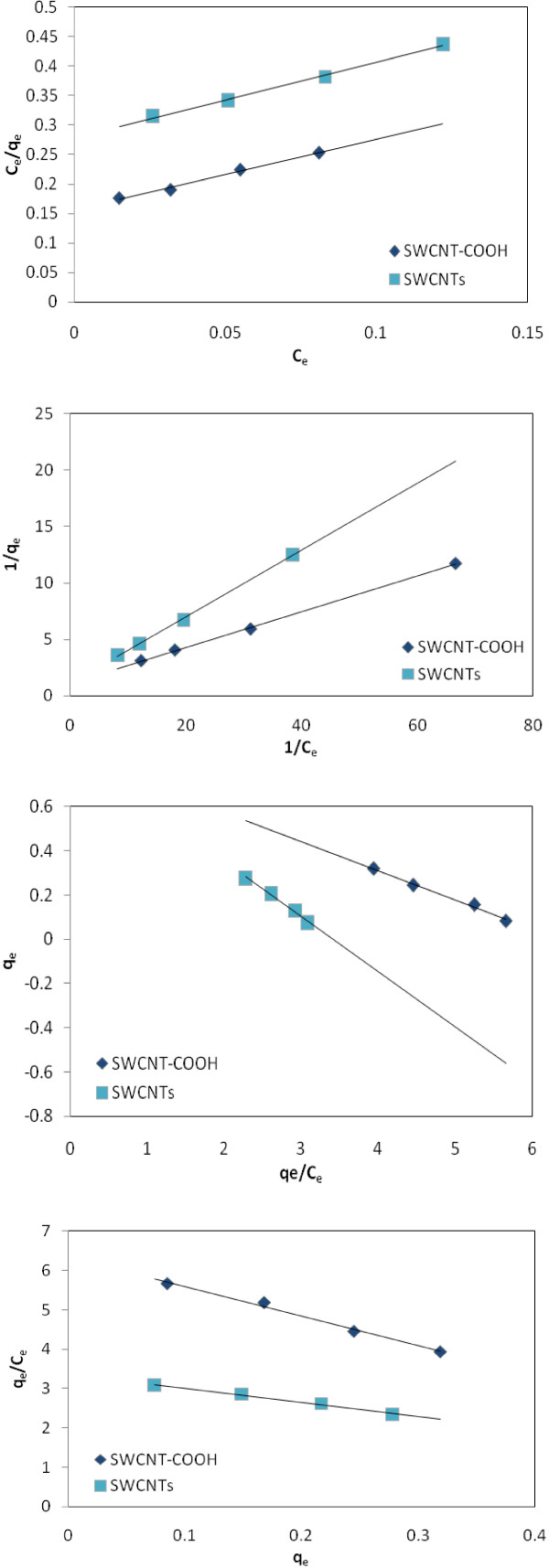
**Langmuir isotherm of EtBr on SWCNTs and SWCNT-COOH; Type 1 (C**_**e**_**/q**_**e**_ ***vs. *** **C**_**e**_**), Type 2 (1/q**_**e**_ ***vs. *** **1/C**_**e**_**), Type 3 (q**_**e**_ ***vs. *** **q**_**e**_**/C**_**e**_**), and Type 4 (q**_**e**_**/C**_**e**_ ***vs. *** **q**_**e**_**).**

The R_L_ value indicates the type of the isotherm to be either irreversible (R_L_ = 0), favorable (0 < R_L_ <1), linear (R_L_ =1) or unfavorable (R_L_ >1) [37]. Figures 1 and 2 shows the adsorption isotherm Langmuir of EtBr on SWCNTs and SWCNT-COOH surfaces, respectively.

The Freundlich model assumes a heterogeneous adsorption surface with sites that have different energies of adsorption and are not equally available [38-40]. The Freundlich isotherm is more widely used but provides no information on the monolayer adsorption capacity, in contrast to the Langmuir model. Its linearized form can be written as:

(5) $$\begin{array}{cc} {\mathrm{lnq}}_{e}={\mathrm{lnK}}_{F}+1/n & {\mathrm{lnC}}_{e} \end{array}$$

where K_F_ (1/mg) and n (dimensionless) are the Freundlich adsorption isotherm constants, being indicative of the extent of adsorption and the degree of nonlinearity between solution concentration and adsorption, respectively.

Temkin isotherm [41] describes the behavior of adsorption systems on a heterogeneous surface, and the linear form of Temkin isotherm is expressed as:

(6) $$q_{e}=\mathrm{\beta ln}K_{T}+\mathrm{\beta ln}C_{e}$$

The adsorption data were analyzed according to Eq. (6). K_T_ is the equilibrium binding constant (L/mg) corresponding to the maximum binding energy and constant b = RT/β (KJ/mol) is related to the heat of adsorption.

Redlich and Peterson [42] incorporate three parameters into an empirical isotherm. The Redliche Peterson isotherm has a linear dependence on concentration in the numerator and an exponential function in the denominator. It approaches the Freundlich isotherm at high concentration and is in accordance with the low concentration limit of the Langmuir equation. Furthermore, the R-P equation incorporates three parameters into an empirical isotherm and, therefore, can be applied either in homogenous or heterogeneous systems due to the high versatility of the equation. It can be described as follows:

(7) $$q_{e}=\frac{K_{R}C_{e}}{1+a_{R}C_{e}^{\beta }}$$

where K_R_ is R-P isotherm constant (L*/*g), a_R_ is R-P isotherm constant (1*/*mg) and *β* is the exponent which lies between 1 and 0. That is, the Henry’s Law equation. Eq. 7 can be converting to a linear form by taking logarithms:

(8) $$\ln\left(K_{R}\frac{C_{e}}{q_{e}}-1\right)={\mathrm{lna}}_{R}+{\mathrm{\beta lnC}}_{e}$$

Therefore a minimization procedure is adopted to maximize the coefficient of determination, between the theoretical data for predicted from the linearized form of Redlich-Peterson isotherm equation and the experimental data.

### Kinetics study

#### Pseudo first-order kinetics

The pseudo first-order equation (Lagergren’s equation) describes adsorption in solid–liquid systems based on the sorption capacity of solids [43]. The linear form of pseudo first-order model can be expressed as:

(9) $$\log\left(q_{e}-q\right)=\log\left(q_{e}\right)-k_{1}t$$

where q_e_ (mg*/*g) and q are the amount each of EtBr adsorbed on the adsorbents at equilibrium and at various times t and k_1_ is the rate constant of the pseudo first-order model for the adsorption (1/min) [43].

#### Pseudo second-order kinetics

The pseudo second-order rate expression, which has been applied for analyzing chemisorption kinetics from liquid solutions [44], is linearly expressed as:

(10) $$t/q=1/k_{2}{q_{e}}^{2}+t/q_{e}$$

where q_e_ and q are defined as in the pseudo first-order model and k_2_ is the rate constant of the pseudo second-order model for adsorption (g*/*mg min) [45].

## Results

### Characterization of carbon nanotubes

**Figure 3:**
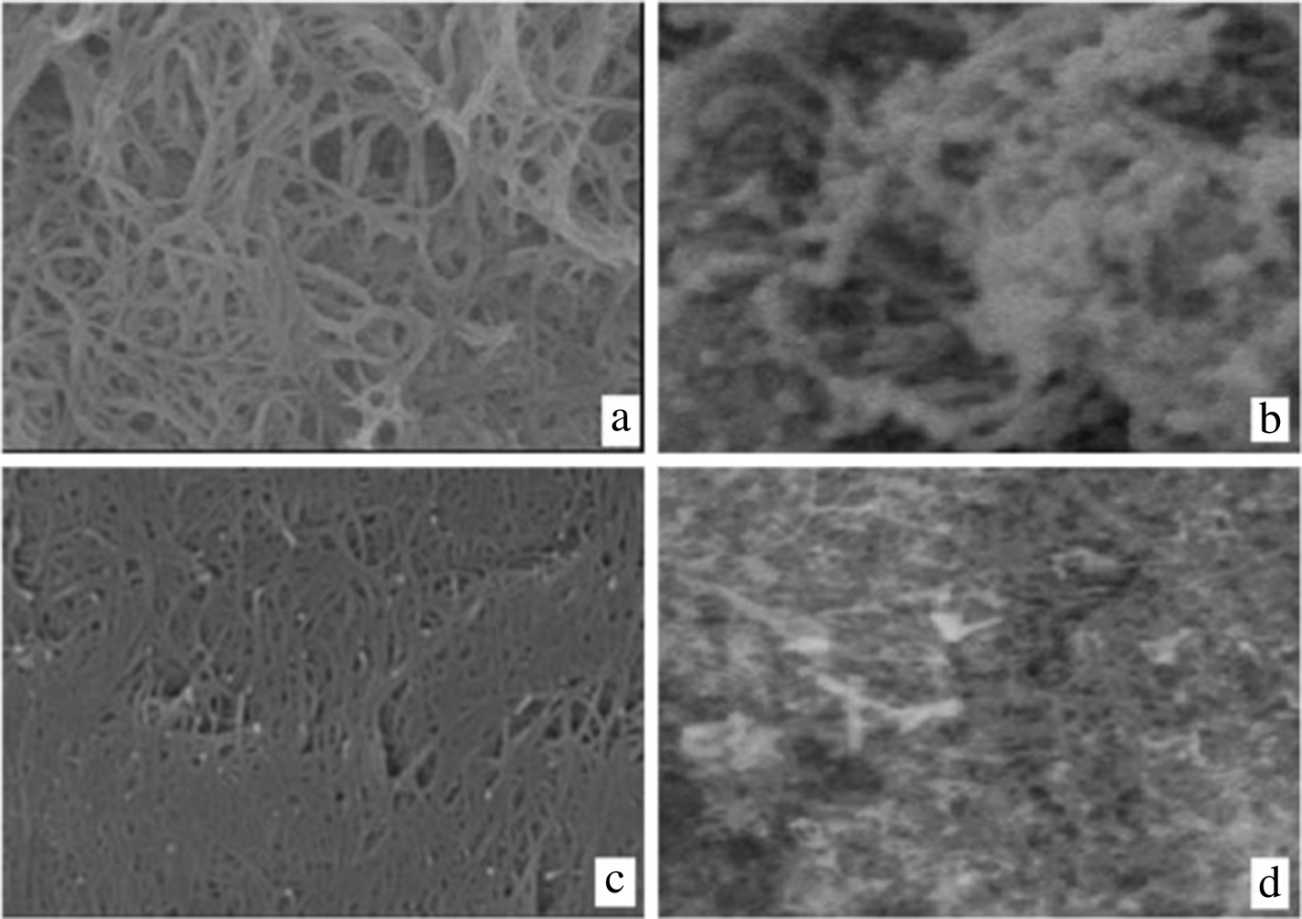
SEM images of SWCNT-COOH (a) before adsorption, (b) after adsorption, and SWCNTs (c) before adsorption, (d) after adsorption.

Figure 3 show the scanning electron microscopy images (SEM) of the carbon nanotubes. As seen in Figure 3 is carboxylate group functionalized single-walled carbon nanotube having a negative surface charge and porosity much more than single-walled carbon nanotube. Based on these textural characteristics explained above it is expected that SWCNT-COOH would present higher sorption capacity than the SWCNTs for the EtBr adsorption, besides of presenting a faster kinetic.

### Effect of contact time

**Figure 4:**
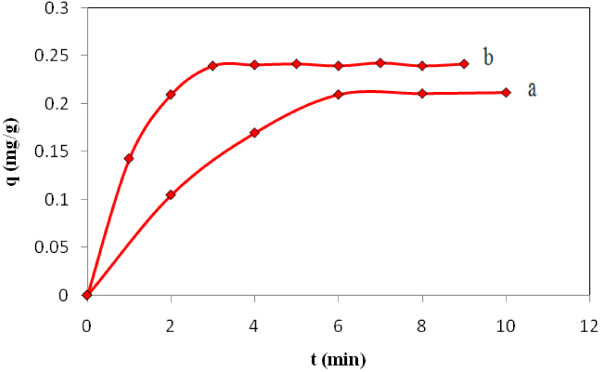
**Effect of contact time on the adsorption of EtBr with (a) SWCNTs and (b) SWCNT-COOH, initial concentration, 20 ml, 30 mg** ***/*** **L; adsorbent dosage, 20 mg and T = 298 ± 1 K.**

Effect of contact time on EtBr adsorption by SWCNT-COOH and SWCNTs were studied by variation of the contact time (0 to 10 min) for constant initial concentrations (30 mg/L). Figure 4 shows the effect of contact time on the adsorption of EtBr on to (a) SWCNTs and (b) SWCNT-COOH.

### Effect of EtBr initial concentration

In the present study, the adsorption experiments are performed to study the effect of EtBr initial concentration by varying it from 10 to 40 mg/L, while maintaining the SWCNT-COOH and SWCNTs amount 0.02 g/L.

### Effect of temperature on the adsorption

Figure 2 shows the representative plots of adsorption amount of EtBr onto SWCNT-COOH and SWCNTs versus different temperature ranging from 298 to 328°K. In this section, concentration of EtBr was 30 mg/L and contacts time were 3 and 6 min for SWCNT-COOH and SWCNTs, respectively. It was found that the adsorption capacity of EtBr onto carbon nanotubes was found to increase with a rise in temperature.

### Equilibrium

**Figure 5:**
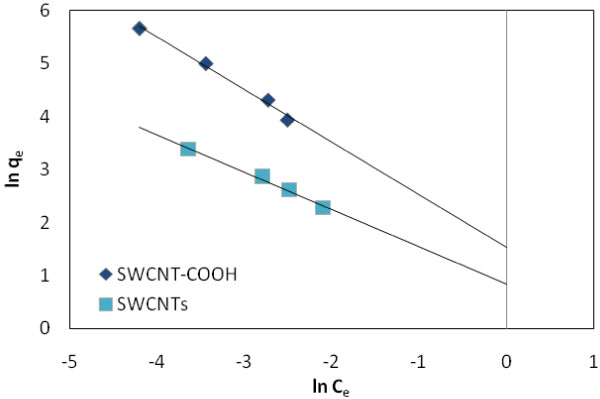
Freundlich adsorption isotherm of EtBr on SWCNTs and SWCNT-COOH surface.

**Figure 6:**
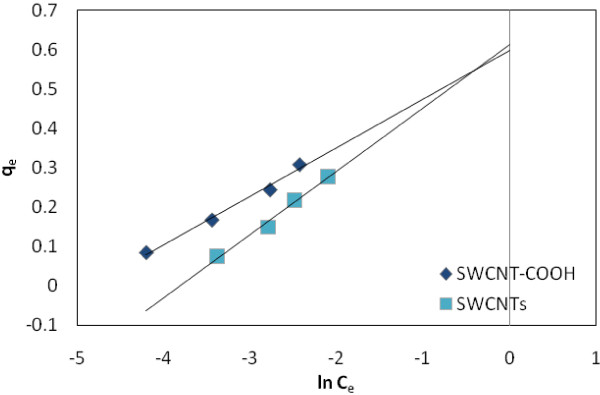
Temkin adsorption isotherm of EtBr on SWCNTs and SWCNT-COOH surface.

**Figure 7:**
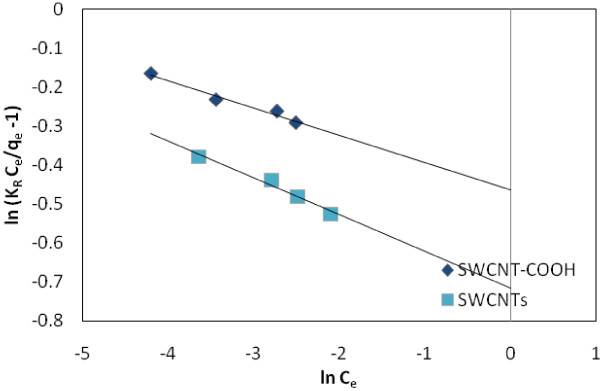
Redlich-Peterson adsorption isotherm of EtBr on SWCNTs and SWCNT-COOH surface.

**Figure 8:**
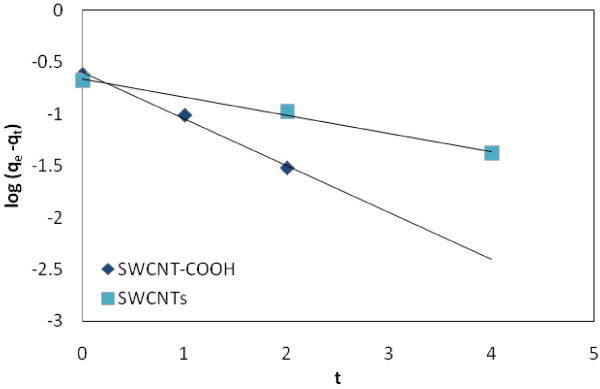
Pseudo first-order kinetics for adsorption of EtBr on SWCNTs and SWCNT-COOH surface.

Figure 5 shows the adsorption isotherm Freundlich of EtBr on SWCNT-COOH and SWCNTs surfaces, respectively. Tables 1, 2 and 3 summarize the coefficients of Langmuir, and Freundlich isotherms for adsorbents, respectively. Figure 6 shows the effect of temperature on the adsorption of EtBr with (a) SWCNTs and (b) SWCNT-COOH. Figures 7 and 8 show the adsorption isotherm Temkin and Redlich-Peterson of EtBr on the adsorbents surfaces, respectively.

**Table 1:** Langmuir isotherm parameters and ARE parameter for EtBr by SWCNTs surface

**Type**	**Equation**	**Q**_**m**_**(mg/g)**	**K(L/mg)**	**R**^**2**^	**R**_**L**_	**ARE**
Type 1	Ce/qe = 1/KQ_m_ + Ce/Q_m_	0.770	4.672	0.9964	0.3-0.6	0.075
Type 2	1/qe = 1/Q_m_ + 1/KQ_m_Ce	0.750	4.555	0.9994		
Type 3	qe = Q_m_–qe/KCe	0.802	4.032	0.9913		
Type 4	qe/Ce = KQ_m_ - Kqe	0.808	4.085	0.9925		

**Table 2:** Langmuir isotherm parameters and ARE parameter for EtBr by SWCNT-COOH surface

**Type**	**Equation**	**Q**_**m**_**(mg/g)**	**K(L/mg)**	**R**^**2**^	**R**_**L**_	**ARE**
Type 1	Ce/qe = 1/KQ_m_ + Ce/Q_m_	0.830	7.751	0.9954	0.2-0.5	0.05
Type 2	1/qe = 1/Q_m_ + 1/KQ_m_Ce	0.844	7.601	0.9958		
Type 3	qe = Q_m_–qe/KCe	0.834	7.633	0.9927		
Type 4	qe/Ce = KQ_m_ - Kqe	0.840	7.553	0.9919		

**Table 3:** Freundlich, Temkin and Redlich-Peterson isotherm parameters for removal EtBr by SWCNT-COOH and SWCNTs surface

**Freundlich**	**K**_**F**_**(mg/g)**		**n**	**R**^**2**^	**ARE**
SWCNT-COOH	4.6089		1.006	0.9903	0.1
SWCNTs	2.3189		1.419	0.9904	0.1
Temkin	K_T_ (L/g)	β (mg/L)	b (KJ/mol)	R^2^	ARE
SWCNT-COOH	126.97	0.1235	20.061	0.9894	0.4
SWCNTs	45.150	0.1608	15.407	0.9882	0.4
Redlich-Peterson	a_R_(l*/*mg)	K_R_(L*/*g)	*β*	R^2^	ARE
SWCNT-COOH	0.9546	11.286	0.0705	0.9728	0.55
SWCNTs	0.4885	4.467	0.0944	0.9732	0.54

### Kinetics

**Figure 9:**
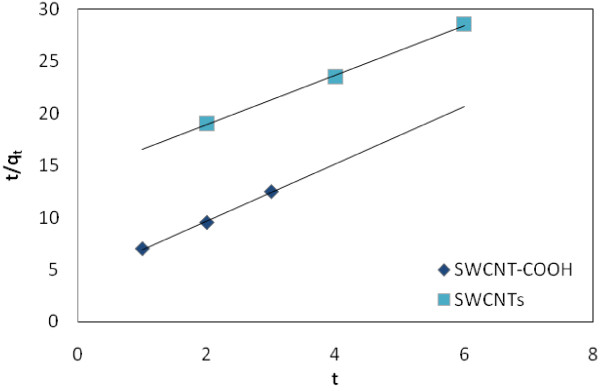
Pseudo second-order kinetics for adsorption of EtBr on SWCNTs and SWCNT-COOH surface.

Figure 9 shows a plot of linearization form of pseudo first-order model. This Fig. shows a plot of linearization form of pseudo second-order model at constant concentrations studied the results of are presented in Table 4.

**Table 4:** Comparison of the pseudo first- and second-order rate constants

	***Pseudo first-order***				***Pseudo second-order***			
	k_1_	q_e_	R^2^	ARE	k_2_	q_e_	R^2^	ARE
	(1/min)	(mg/g)			(g*/*mg min)	(mg/g)		
SWCNT-COOH	0.1755	0.281	0.9946	0.2	7.6335	0.266	0.9978	0.1
SWCNTs	0.4515	0.238	0.9933	0.2	5.7142	0.230	0.9988	0.1

## Discussion

### Effect of contact time

According to Figure 4, EtBr adsorption rate increased quickly with time and then reached equilibrium. SWCNT-COOH and SWCNTs surfaces were treated by EtBr solutions (30 mg*/*L and T = 298 ± 1 K) in order to optimize contact time respect to EtBr. The amounts of adsorbed ion on the adsorbents were analyzed using UV-visible Spectrophotometer.

### Effect of temperature

According to Figure 1; the adsorption capacity of EtBr onto carbon nanotubes was found to increase with a rise in temperature, suggesting the process in this research has been endothermic [44]. An increase in the amount of equilibrium adsorption of each ion with the rise in temperature may be explained by fact that the adsorbent sites were more active at higher temperatures.

### Adsorption isotherms

According to Figure 2 the constants of Langmuir models is obtained from fitting the adsorption equilibrium data and is listed in Tables 1 and 2. A comparison of the R^2^ and ARE values given in Tables 1 and 2 with 2 indicates that Langmuir isotherm better fits the experimental data than does the other isotherms. The validity of Langmuir isotherm suggests that adsorption is a monolayer process, and adsorption of all species requires equal activation energy. Moreover, K values for various adsorbents followed the order SWCNT-COOH *>* SWCNTs, suggesting that the affinity of the binding sites for each ion also followed this order. The R_L_ parameter lies between 0.22 and 0.60 which proves that the adsorption process is favorable and SWCNTs and SWCNT-COOH are potential adsorbents for the removal of EtBr from drinking water.

### Adsorption Kinetics

The high correlations coefficient and high agreement that exist between the calculated and experimental *q*_e_ values of the pseudo second-order kinetic model over the other model renders it best in adsorption of EtBr. This confirms that the sorption data for removal of EtBr on SWCNTs and SWCNT-COOH are well represented by the pseudo-second-order kinetics for the entire sorption period.

### Reusability

Repeated usage of CNT filters is one of the critical considerations in a treatment plant from economics standpoint. CNT filters are reusable as evidenced by Brady-Estevez et al. (2008) and Srivatsava et al. (2004). On the other hand CNT filters, due to their excellent mechanical properties prevent such deformational changes. Moreover, CNT filters support simple thermal regeneration techniques, whereas with polymeric membranes it is not possible [46,47].

## Conclusion

This investigation examined the equilibrium and the dynamic adsorption of EtBr on SWCNT-COOH and SWCNTs surfaces. The adsorption capacity was highest when 20 mg*/*l SWCNT-COOH and SWCNTs were added. The results suggested that the adsorption of EtBr on SWCNT-COOH and SWCNTs surfaces increased with temperature. The adsorption of mentioned ions on SWCNT-COOH is more than SWCNTs surfaces. SWCNT-COOH and SWCNTs are after adsorption can be recycle and usable. In addition, SWCNT-COOH and SWCNTs are adsorbents have desorption capabilities.

## Competing interests

The authors declare that they have no competing interests.

## Authors’ contributions

MN and KN carried out the mechanism of adsorption studies and participated in the drafted the manuscript. OM and AF carried out the isotherms and kinetics of adsorption studies and the effect of different parameters studies. All authors read and approved the final manuscript.
